# Generation and Characterization of a Genetically Modified *Zea mays* Line with a Knockdown of Hypoxia-Dependent microRNA775A

**DOI:** 10.3390/ijms27072943

**Published:** 2026-03-24

**Authors:** Dmitry N. Fedorin, Anna E. Khomutova, Alexander T. Eprintsev, Abir U. Igamberdiev

**Affiliations:** 1 Department of Biochemistry and Cell Physiology, Voronezh State University, 394018 Voronezh, Russia; rybolov@mail.ru (D.N.F.); anna.khomutova2002@gmail.com (A.E.K.); bc366@bio.vsu.ru (A.T.E.); 2 Department of Biology, Memorial University of Newfoundland, St. John’s, NL A1C 5S7, Canada

**Keywords:** *Zea mays*, *Agrobacterium tumefaciens*, aldolase, hypoxia, microRNA775A, gene knockdown

## Abstract

Hypoxia-dependent microRNAs play an important role in orchestrating a plant’s response to low-oxygen stress. To assess the regulatory mechanisms of the adaptive response of maize (*Zea mays* L.) to hypoxia, an antisense sequence was developed, and the short tandem target mimic (STTM) system was used to induce the loss of function of the mature microRNA775A (miR775a) in maize. A recombinant binary vector pBI121 cloned in *E. coli* cells containing the antisense sequence anti-miR775A to maize miR775A was acquired to create a line of modified *A. tumefaciens EHA105*. Using the puncturing method on soaked seeds, maize plants with an active anti-miR775A construct were obtained, as evidenced by a decrease of more than 10-fold in mature miR775A content and by developmental changes in the seedlings. The size of seedlings of the maize knockdown line was almost twice smaller than that of the wild-type (WT) plants. An assessment of the effects of hypoxic conditions induced by flooding of 14-day-old maize plants revealed differences in the expression and activity of several enzymes between WT and knockdown plants. The reduced miR775A levels led to a 2.1-fold drop in pyruvate levels, which resulted in decreased pyruvate kinase, pyruvate dehydrogenase, and lactate dehydrogenase activities as compared to WT plants. A decrease in miR775A content in the maize knockdown cell line also affected the function of mitochondrial and extramitochondrial isoenzymes of citrate synthase, aconitase, and fumarase under hypoxic conditions.

## 1. Introduction

Small non-coding RNAs play an important role in the regulation of metabolism in plants, animals, and fungi [1,2]. To maintain cell homeostasis under stress conditions, plants employ several mechanisms [3], including regulating the transcription of hypoxia-sensitive genes and post-transcriptionally regulating gene expression by binding to messenger RNA (mRNA) and either inhibiting protein synthesis or triggering mRNA degradation. At low oxygen concentrations (below 2%), essential adaptive responses are triggered through microRNAs (miRNAs) [4], which are the endogenous small non-coding RNAs that regulate the expression of target genes at the post-transcriptional level. A large number of plant miRNAs are involved in abiotic and biotic stresses, acting through RNA silencing, including miR775A, whose induction has been demonstrated under hypoxia [4].

The discovery of miRNAs has provided new insights into cellular regulatory processes. Several studies show that perfectly matched miRNAs are neither necessary nor sufficient for detecting all functional miRNA-target interactions [5,6]. Most plant miRNAs exhibited perfect or near-perfect complementarity to their target mRNAs, but complementarity may not be the sole determining factor for effective protein translation suppression. Furthermore, several plant miRNAs, such as *Arabidopsis* miR398, have multiple targets [6]. Due to the partial overlap of the roles of various coexpressed miRNAs, functional studies of a specific miRNA can best be achieved by knocking down the corresponding miRNA. This technology was made possible by the discovery of the target mimic *INDUCED BY PHOSPHATE STARVATION1 (IPS1)* in *Arabidopsis* [7] and was subsequently developed into a powerful technology called Short Tandem Target Mimic (STTM) [8]. STTMs contain a single pair of miRNA-binding motifs connected by a 48-nucleotide spacer, which forms a short stretch of unpaired nucleotides, thereby increasing the stability of STTM structures. The complete complementarity between miRNAs and artificial miRNA binding sites, as well as the stability of STTMs, enables this technology to knock down any miRNA, resulting in loss of function [9].

Many plant miRNA targets are believed to play an important role in plant adaptation to hypoxic conditions [10]. Nineteen families of hypoxia-related miRNAs have been identified [11]. MiRNA775A (miR775A) is a representative of the hypoxia-dependent miRNA family in plant cells [4], one of the targets of which is *GALACTOSYLTRANSFERASE 9 (GALT9)*. Proteins of the GALT family are involved in cell wall remodelling [12]. MiRNA775A is directly repressed by the transcription factor ELONGATED HYPOCOTYL 5 (HY5). HY5 is a key regulator of photomorphogenesis, binding to light-responsive elements in promoters that regulate *MICRORNA* (*MIR*) genes. Genetic analysis confirmed that HY5 negatively regulates leaf size through HY5-miRNA775A-GALT9 [13].

The glycerol-3-phosphate acyltransferase gene (*Z. mays*, LOC100285746) has also been identified as a target of miRNA775A [14,15]. Members of this protein family include several N-acetyltransferases (GNATs): aminoglycoside N-acetyltransferases, histone N-acetyltransferases (HATs), serotonin N-acetyltransferases, arginine/ornithine N-succinyltransferase, etc. [16]. Glycerol-3-phosphate acyltransferase is involved in the metabolism of glycerophospholipids and phosphoglycerides, the alpha-glycerophosphate pathway. Polyunsaturation of long-chain acyl-CoA regulates hypoxia sensitivity in plants by modulating the dynamics of acyl-CoA-binding protein [17]. Lipids also mediate signalling during plant responses to biotic and abiotic stress [18,19]. The involvement of miRNAs in plant responses to hypoxia was further confirmed by experiments in which mitochondrial respiration was inhibited [4].

In a previous study, we showed that miR775A regulates the expression of two aldolase genes that are important in the adaptive response of cellular metabolism to hypoxia [20]. It was previously established that miR775A regulates the aldolase reaction of glycolysis during hypoxia via RNA interference. It forms an RNA-interfering complex with the major isoenzyme of cytoplasmic fructose-1,6-bisphosphate aldolase, thereby reducing its transcript levels during hypoxia [20]. Under oxygen-deficient conditions, plant cells experience an energy crisis because they cannot use the classical electron transport chain. As a result, glycolysis becomes the primary metabolic pathway, partially compensating for the ATP deficit. Therefore, studying miR775A is relevant to its role in regulating plant metabolism under hypoxic conditions.

Understanding the role of miR775A in the regulation of energy and constructive metabolism is crucial for assessing its significance in plant adaptation to hypoxia. The observed lack of coordination between the isoenzyme composition of maize aldolase under hypoxic stress, mediated by miR775A [20], may have significant implications for plant adaptation. Induction of the ALDO2 isoenzyme in maize leaves during hypoxia helps maintain the required level of glycolysis; however, a decrease in miR775A levels under hypoxia may negatively affect the cellular metabolic response.

A true assessment of the contribution of miR775A to the regulation of adaptive responses is possible by creating a mutant plant line with reduced levels of the corresponding mature miRNA. *A. tumefaciens*-mediated plant transformation is the most common method for transforming monocots and dicots [21]. This study aimed to generate a modified *A. tumefaciens* EHA105 strain containing the antisense sequence anti-miR775A and to obtain a knockdown line of *Z. mays* plants with reduced levels of mature miR775A to elucidate its function in plant metabolism and adaptation to hypoxic stress.

## 2. Results

### 2.1. Generation of a Z. mays Plant with a miR775A Knockdown

*A. tumefaciens EHA105*, containing the pBl121 vector with the antisense anti-miR775A sequence, was used to generate a *Z. mays* plant line with appropriate miR775A knockdown using a seed piercing protocol. The anti-miR775A construct for *Zea mays* consists of two non-cleavable binding sites for miRNA775A with a three-nucleotide insertion between the 10th and 11th nucleotides, connected by a 48-nucleotide spacer, which is a loop region (Supplementary Figure S1). BamH1 restriction sites are incorporated at the 5′ and 3′ ends. Five additional nucleotides are also present at the 5′ and 3′ ends, which are necessary for the successful use of specially designed primers to generate double-stranded anti-miR775A during the polymerase chain reaction.

PCR analysis of maize DNA from all three lines for the presence of the pBl121 vector using M13 primers showed that the amplicons were obtained only from DNA samples from WT-Ab maize plants modified with the original pBl121 vector without the anti-miR775A construct and from mir775A knockdown maize plants (Supplementary Figure S2). The amplification products using M13 primers and the anti-miR775A construct indicate that the maize transformation method yielded plants containing pBl121 vector with the anti-miR775A construct.

An analysis of the identification of the anti-miR775A construct used for maize transformation revealed the presence of an amplification product with specific primers only in the DNA sample isolated from miR775A knockdown plants (mir775A). The size of the PCR product was within the theoretical value, indicating the presence of pBl121 vector with the anti-miR775A construct in maize cells (Supplementary Figure S3).

**Figure 1:**
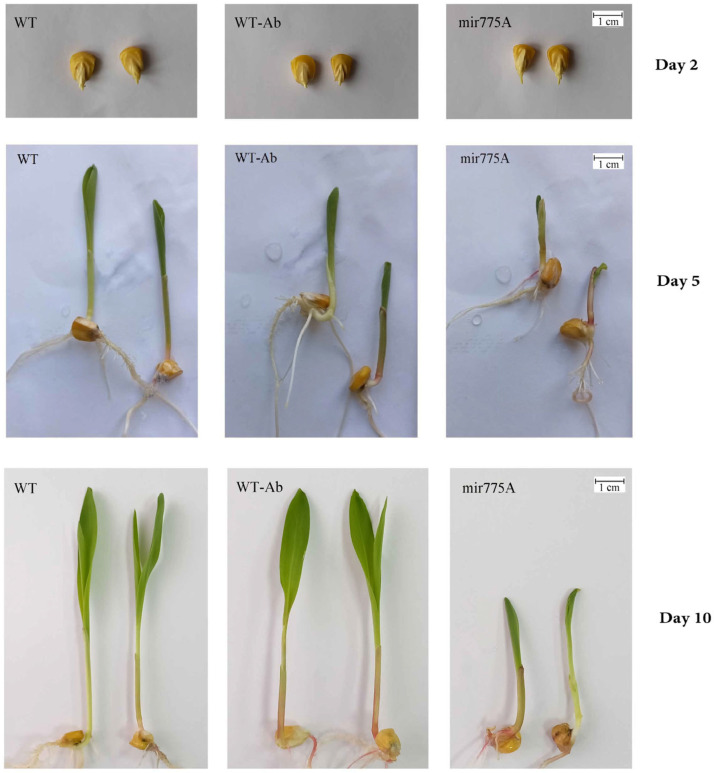
Phenotypes of wild-type (WT) maize plants, the plants modified with the original *A. tumefaciens EHA105* line (WT-Ab), and miR775A knockdown (mir775A) plants at different days of germination.

The resulting miR775A-knockdown *Z. mays* lines were morphologically distinct from the WT plants and from the plants modified with the empty (not containing pBl121 vector with the anti-miR775A) *A. tumefaciens EHA105* (WT-Ab). Plants with miR775A knockdown demonstrated reduced coleoptile development and leaf blade expansion compared to controls during seedling formation and development (Figure 1).

Compared to the control WT and to WT-Ab plants, the length of the mir775A line seedlings is 1.82 and 1.76 times shorter, respectively (Table 1).

**Figure 2:**
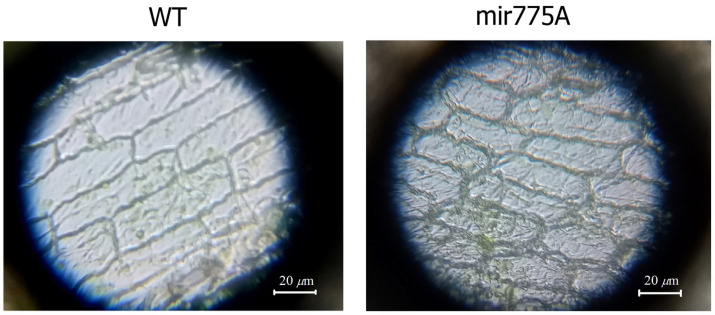
Photographs of wild-type (WT) and mir775A maize leaf epidermal cells obtained using an Olympus Cover-015 microscope (Olympus, Japan) at 400× magnification.

The size of meristem cells in maize leaves was compared using microscopy. The analysis was performed using an Olympus Cover-015 microscope (Olympus, Shinjuku, Tokyo, Japan) at 400× magnification. Figure 2 shows an approximately 1.5-fold smaller cell size in mir775A plants. The smaller size of epidermal cells in mir775A maize leaves is due to the involvement of mature miRNA775A in the control of GALT family proteins, which may be associated with impaired functioning of GALT9, which is responsible for cell wall modelling and elongation [12]. It has previously been shown that in Arabidopsis plants with miRNA775 overexpression, leaf size increased due to larger cell size [12].

**Figure 3:**
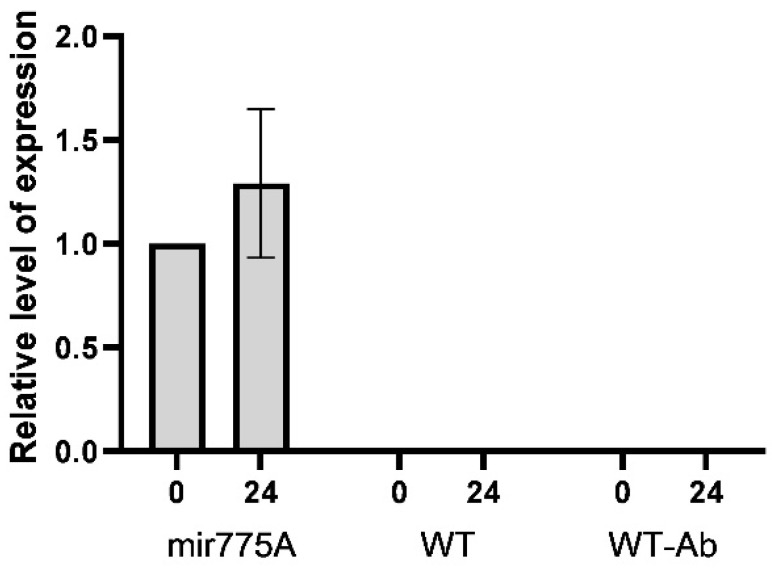
Anti-miR775A expression in three plant lines. WT—wild-type maize plants, WT-Ab—maize plants modified with the original *A. tumefaciens* EHA105 line, and mir775A—maize plants with suppressed expression. Experiments were performed in three biological and four analytical replicates. Briefly, 0 and 24 h of hypoxia were used. Analysis of variance (ANOVA) was used to process the results.

It was shown that the miR775A line expresses the anti-miR775A construct, while no expression was detected in WT plants or those transformed with empty Agrobacterium (Figure 3). When miR775A maize plants were exposed to hypoxia for 24 h, no significant difference was observed in the transcript levels of the anti-miR775A construct, indicating the stability of its expression.

**Figure 4:**
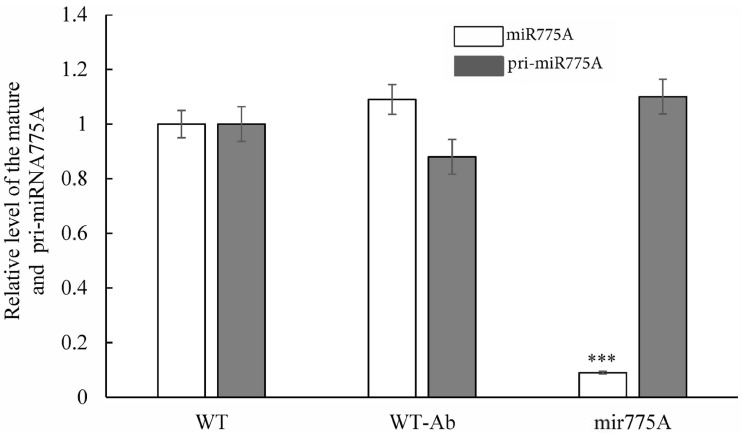
Relative levels of miR775A (white bars) and its precursor (pri-miR775A, grey bars) in the leaves of control and experimental maize samples. WT—wild-type maize plants, WT-Ab—maize plants modified with the original *A. tumefaciens EHA105* line, mir775A—knockdown maize plants. Asterisks (***) indicate significant differences from WT (*p* < 0.005). Analysis of variance (ANOVA) was used to process the results. Experiments were conducted in three biological and four analytical replicates.

Using real-time PCR, we determined miRNA775A levels in the leaves of WT, WT-Ab, and mir775A plants (Figure 4). Compared to the WT and WT-Ab control groups, the amount of free miRNA775A in mir775A leaves was reduced by 11-fold. Furthermore, the levels of the miRNA775A precursor (pri-miRNA) were stable across all studied plants, indicating that the corresponding gene’s transcriptional activity remained unchanged.

The use of the anti-miR775A construct and *Agrobacterium*-mediated transformation made it possible to obtain the knockdowns of maize plants with reduced levels of mature miR775A.

### 2.2. Effects of Hypoxia and miR775A on the Expression of Aldolase Genes and Aldolase Activity

**Figure 5:**
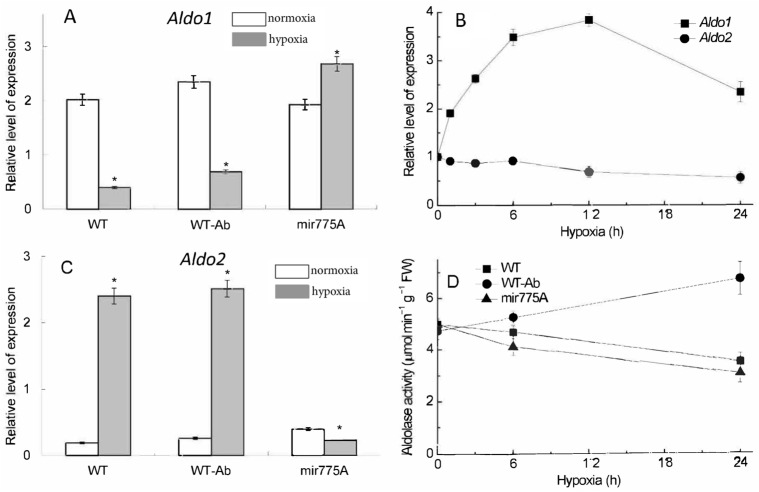
Influence of hypoxia and miR775A expression on the expression (**A**–**C**) and activity (**D**) of aldolase. (**A**)—Relative level of the *Aldo1* gene, and (**B**)—changes in expression of the *Aldo1* and *Aldo2* genes in mir775A plants during the hypoxic incubation. (**C**)—Relative level of the *Aldo2* gene; white bars—normoxia, grey bars—24 h hypoxia; asterisks (*) indicate significant differences from the normoxic values (*p* < 0.05). Analysis of variance (ANOVA) was used to process the results. (**D**)—Aldolase activity during the hypoxic incubation in the WT, WT-Ab and mir775A plants. Experiments were conducted in three biological and four analytical replicates.

The transcript levels of the two genetically determined cytosolic isoenzymes of the glycolytic enzyme fructose-1,6-bisphosphate aldolase revealed major differences between the two genes. Expression of the *Aldo1* gene was strongly suppressed after 24 h of hypoxia in the WT and WT-Ab plants (Figure 5A), while *Aldo2* expression was strongly activated by hypoxia (Figure 5C). The knockdown of miR775A resulted in a noticeable increase in *Aldo1* expression and a decrease in *Aldo2* expression (Figure 5A,C). During hypoxic incubation, Aldo1 expression gradually increased, peaking at 12 h of hypoxia and then declining. In comparison, Aldo2 expression decreased slightly but gradually over 12–24 h of hypoxia (Figure 5B).

Measurements of fructose-1,6-bisphosphate aldolase activity in leaves of WT, WT-Ab and mir775A plants under normoxia and after 24 h of hypoxia revealed that during the first 6 h of hypoxia, no significant deviations from control values (0 h) were observed in WT and WT-Ab plants. In contrast, the activity decreased by 1.2 times in mir775A plants. After 24 h of hypoxia, activity further decreased in the mir775A line and WT, whereas in WT-AB plants it markedly increased (Figure 5D).

Reduced content of mature miR775A in mir775A knockdown maize lines contributes to a decrease in aldolase enzymatic activity relative to WT plants.

### 2.3. Effects of Hypoxia and miR775A on the Expression of the Genes Encoding Respiratory Enzymes

**Figure 6:**
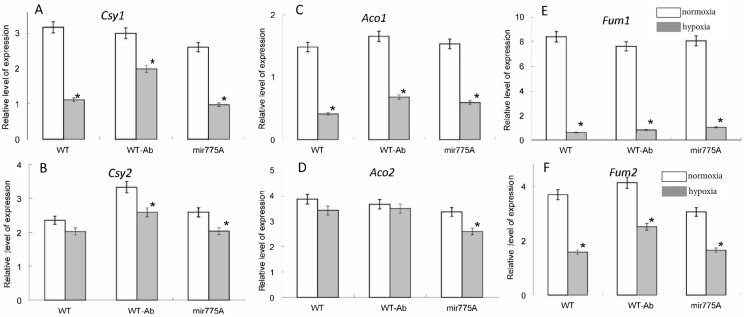
Influence of hypoxia and miR775A expression on the expression of the genes encoding mitochondrial (**upper row**) and extramitochondrial (**lower row**) respiratory enzymes. (**A**,**B**)—*Csy1* and *Csy2*—the genes encoding correspondingly the mitochondrial and the peroxisomal forms of citrate synthase; (**C**,**D**)—*Aco1* and *Aco2* –the genes encoding the mitochondrial and the cytosolic aconitase; (**E**,**F**)—*Fum1* and *Fum2*—the genes encoding the mitochondrial and the cytosolic fumarase. White bars—normoxia, grey bars—24 h hypoxia. Asterisks (*) indicate significant differences from the normoxic values (*p* < 0.05). Analysis of variance (ANOVA) was used to process the results. Experiments were conducted in three biological and four analytical replicates.

Expression of the genes encoding the mitochondrial isoforms of citrate synthase, aconitase, and fumarase in WT plants was strongly suppressed by hypoxia. At the same time, downregulation of miR775A had no significant effect compared to WT (Figure 6A–C). For the gene encoding the peroxisomal form of citrate synthase, the effect of hypoxia was observed only in WT-Ab and mir775A plants, and it was very weak. For the gene encoding cytosolic aconitase, a very weak effect (20% inhibition) was observed only in mir775A plants, while expression of the cytosolic fumarase gene was suppressed twofold in all lines (Figure 6D–F).

Under hypoxic conditions, less pronounced changes in the transcriptional activity of the genes encoding the extramitochondrial isoenzymes citrate synthase, aconitase, and fumarase were demonstrated in WT plants. In mir775A plants, the inhibition of these isoenzymes was more significant compared to WT plants.

Figure 6 shows the expression profiles of TCA cycle isoenzymes with both mitochondrial and extramitochondrial localizations. Evaluation of their transcriptional activity allows us to assess the role of reduced miR775A levels in plant cells in coordinating cellular energy and plastic metabolism. As shown, the pattern of changes in their expression is consistent with that in WT plants, but to varying degrees. This can be explained by a common cellular response strategy to oxygen deficiency, taking into account the Aldo1/Aldo2 isoenzyme ratio, which is regulated by miR775A.

### 2.4. Effects of Hypoxia and miR775A on the Activity of Pyruvate Metabolizing Enzymes and Pyruvate Content

**Figure 7:**
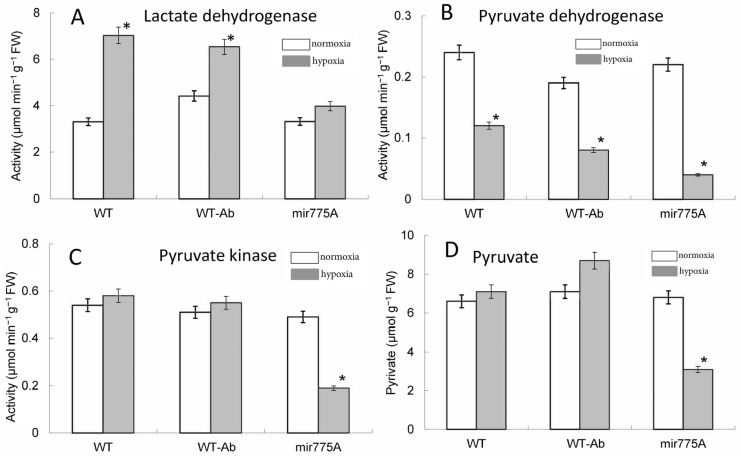
Influence of hypoxia and miR775A expression on pyruvate metabolizing enzymes: lactate dehydrogenase (**A**), pyruvate dehydrogenase (**B**), pyruvate kinase (**C**), and on pyruvate content (**D**) in maize leaves. White bars—normoxia, grey bars—24 h hypoxia. Asterisks (*) indicate significant differences from the normoxic values (*p* < 0.05). Analysis of variance (ANOVA) was used to process the results. Experiments were conducted in three biological and four analytical replicates.

In WT and WT-Ab plants, hypoxia led to an increase in lactate dehydrogenase (LDH) activity and a decrease in pyruvate dehydrogenase (PDH) activity (Figure 7A,B). In plants of the mir775A line, the effect of LDH activation was not observed. The level of PDH activity after 24 h of hypoxia was significantly lower than under normoxia in all lines, but in the mir775A line, the decrease was stronger (Figure 7B). No significant changes in the catalytic activity of pyruvate kinase were detected for the WT and WT-Ab maize lines under normal or hypoxic conditions. In contrast, in the mir775A line, the activity was reduced by more than twice (Figure 7C). The content of pyruvate in the leaves of WT, WT-Ab and mir775A maize lines under normal conditions and after 24 h of hypoxia reveals a decrease only in the plants of the mir775A line (Figure 7D).

Knockdowns of the mir775A maize line show a change in pyruvate metabolism, since a decrease in its content was found due to less intense glycolysis, which causes only a slight increase in the enzymatic activity of LDH.

## 3. Discussion

The miRNA 775A studied belongs to the group of hypoxia-dependent miRNAs and participates in plant adaptation to low-oxygen conditions [4]. Under hypoxia, the corresponding free miRNA accumulates, indicating its involvement in stress response [15]. To study the role of miRNA775A in maize’s adaptive response to hypoxic conditions, Agrobacterium-mediated transformation was used to generate knockdown plants with reduced mature miR775A levels.

The obtained results from the germination assessment of the original and modified maize seeds (Table 1) allowed us to identify phenotype differences. The smaller size of the mir775A maize seedlings is due to the involvement of mature miR775A in the control of GALT family proteins, which modulate cell wall formation, as previously demonstrated [12]. Furthermore, on the tenth day of development, the unfolding of the first leaves was observed in WT seedlings and plants treated with the original *Agrobacterium* with an empty vector pBl121, which was virtually absent in mir775A plants (Figure 1). The morphological differences between the knockdown plants and the control groups may be due to impaired GALT9 function, which is responsible for cell wall modelling and elongation [12] and lipid metabolism [15]. A reduction in the size of epidermal cells in mir775A maize leaves compared to the WT plant (Figure 2) confirms impaired GALT9 function with reduced miR775A levels in the cells, which is consistent with the active function of anti-miR775A in the experimental plant cells. Knockdown plants showed a significant reduction in mature miR775A levels compared to WT and WT-Ab, but levels of its precursor were stable in both WT and mir775A plant cells (Figure 3).

The observed increase in aldolase activity in WT-Ab maize plants may be associated with intensified glycolytic processes in plant cells in the presence of *A. tumefaciens* and with the redirection of metabolites into the shikimate pathway [22].

The increase in *Aldo2* gene transcripts in maize leaves under hypoxia is likely due to the need to synthesize a form of the enzyme that is more functional under hypoxic conditions to maintain the rate of glycolysis, the main source of ATP under oxygen deficiency. This change in the aldolase gene mRNA content in mir775A maize leaves is associated with the presence of an active anti-mir775A system, which ensures the degradation of mature miR775A and, consequently, the absence of an inhibitory effect on the *Aldo1* gene. Maintenance of the glycolytic metabolism under hypoxic conditions requires the activation of Aldo2 isoenzyme and the inhibition of Aldo1, which is absent in knockdowns. This is in a good agreement with our data on the mechanism of suppression of aldolase activity by RNA interference [20]. Therefore, the obtained miR775A knockdown maize plants confirm the importance of this miRNA in the regulation of cytosolic fructose-1,6-bisphosphate aldolase, which plays a crucial role in organizing the glycolytic pathway in maize cells under hypoxia by switching the transcriptional activity of the genes encoding two isoforms of this enzyme.

The data on the activity of pyruvate-metabolizing enzymes and on pyruvate content (Figure 7) revealed that under hypoxic conditions, mitochondrial oxidative metabolism is significantly inhibited, which includes a decrease in the rate of pyruvate metabolism by mitochondria via the PDH complex. The observed increase in LDH activity in WT and WT-Ab plants indicates the intensified fermentation, which is important under hypoxic conditions to maintain glycolysis and regenerate NADH, while in the mir775A plants, the glycolytic flux is inhibited, as seen from the absence of LDH increase. This may be due to the suppression of the *Aldo2* isoenzyme in the mir775A plants. The Aldo1 isoenzyme is likely less tolerant to hypoxic conditions, exhibiting reduced catalytic activity, and pyruvate levels in mir775A plants are lower than in WT. Consequently, there is no change in LDH activity in mir775A plants. The results of measuring the activity of PDH complex (Figure 7B) and of the expression of citrate synthase, aconitase and fumarase (Figure 6) confirm that hypoxia has a pronounced effect on mitochondrial metabolism, which is primarily due to a lack of oxygen for the operation of the mitochondrial respiratory chain. The effect of miR775 downregulation on the activity of the pyruvate metabolizing enzymes and on the expression of the cytosolic form of aconitase is minor as compared to the effect on aldolase isozymes, and can be an indirect consequence of the alteration in glycolytic metabolism caused by this miRNA. The lack of reorganization of the Aldo1 and Aldo2 isoenzymes in hypoxic knockdown maize plants leads to reduced pyruvate production, which affects LDH activity and the need for intensive NADH regeneration. Consequently, the lack of coordination between Aldo1 and Aldo2 in the mir775A plant line is a key parameter in the control of the glycolytic pathway during hypoxia, a key component of the adaptive response of cellular metabolism.

**Figure 8:**
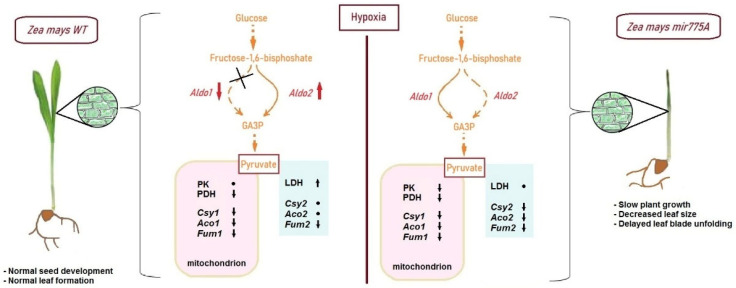
Hypothetical scheme of the role of miR775A in the regulation of hypoxic stress in leaves of WT and miR775A knockdown maize. PK, pyruvate kinase; LDH, lactate dehydrogenase; *Csy1*, *Csy2*, the genes encoding the mitochondrial and peroxisomal citrate synthase isoenzymes; *Aco1*, *Aco2*, the genes encoding the mitochondrial and cytosolic aconitate hydratase isoenzymes; *Fum1*, *Fum2*, the genes encoding the mitochondrial and cytosolic fumarate hydratase isoenzymes. The dash arrow indicates the inhibition, and the crossed dash arrow indicates the suppression of the reaction. Dots indicate no change in enzymatic or transcriptional activity in relation to normoxia; arrows indicate the increase or decrease in enzymatic or transcriptional activity in relation to normoxia.

We conclude that our study of knockdown maize plants with hypoxia-dependent miR775A revealed its involvement in the regulation of glycolytic metabolism. A decrease in the mature miR775A level in knockdown plants leads to a decrease in the rate of glycolysis, manifested by a decrease in pyruvate levels, which occurs due to a decrease in its synthesis in the pyruvate kinase reaction. In WT plants, the switch to *Aldo2* mediated by miR775A maintains pyruvate levels. Activation of LDH with decreased mitochondrial enzyme function is necessary for maintaining cellular energy status and NADH regeneration (Figure 8). In knockdown plants, the Aldo1 isoenzyme, which is likely less tolerant to hypoxia, remains more active, leading to a decrease in glycolytic flux and pyruvate levels. This effect may also be associated with the efflux of phosphoenolpyruvate to the shikimate pathway [22], activated by hypoxia, as evidenced by the reduced pyruvate kinase activity in mir775A plants. Consequently, low pyruvate levels do not promote an increase in LDH activity in knockdown plants. This can be explained by the limitation in switching of the aldolase reaction to the second isoenzyme, which was shown to maintain glycolytic metabolism under low oxygen conditions [20]. Consequently, in knockdown maize plants with hypoxia-dependent miR775A, the intensity of the aldolase reaction is reduced due to dysregulation of the cytosolic isoenzymes encoded by the genes *Aldo1* and *Aldo2* (Figure 8).

## 4. Materials and Methods

### 4.1. Object of Investigation

The seeds of maize (*Zea mays* L., cv. Voronezhskaya-76) were obtained from the Voronezh branch of the All-Russian Research Institute of Maize, Voronezh, Russia. Voronezhskaya-76 is an old, historical maize cultivar bred in the early 1940s at the All-Russian Research Institute of Maize, known for its hardiness and good yield, producing vigorous plants with several ears per stalk. The transformed plants in this study were created by the authors, as described below. Maize plants were grown hydroponically under 12 h of daylight at an intensity of 90 μmol quanta m^−2^ s^−1^ at 25 °C. The leaves of 14-day-old plants were used to monitor the effects of hypoxia on enzyme activities and gene expression.

### 4.2. Construction of Anti-miR775A

To create a specific anti-miR775A construct, STTM technology for the inactivation of endogenous miRNAs in *Arabidopsis* was used [9]. The anti-miR775A construct for *Zea mays* consists of two non-cleavable binding sites for miR775A with a three-nucleotide insertion between the 10th and 11th nucleotides, connected by a 48-nucleotide spacer, which is a loop region. BamH1 restriction sites are incorporated at the 5′ and 3′ ends. Five additional nucleotides are also present at the 5′ and 3′ ends, which are necessary for the successful use of specially designed primers to generate double-stranded anti-miR775A during the polymerase chain reaction (Supplementary Figure S1).

### 4.3. Generation of a Transformed E. coli Line

The antisense construct anti-miR775A, and the pBl121 vector were digested with BamHI restriction endonuclease (SibEnzyme, Russia). The restriction mixture was incubated for 14 h at 37 °C. The antisense construct and vector were purified from the reaction mixture using the Cleanup Standard kit (Eurogen, Moscow, Russia), according to the manufacturer’s protocol, and used for the ligation reaction. The mixture volume was 20 µL, and the reaction was carried out using T4 DNA ligase (Eurogen, Moscow, Russia) at 14 °C for 16 h. The ligation mixture was used to transform competent *E. coli HB101* cells.

The vector was cloned into *E. coli* cells. For this, competent *HB101* cells (100 µL) were transformed with the vector (10 µL) using the heat-shock method according to the standard protocol [23]. Transformants were plated on Petri dishes containing Luria-Bertani (LB) agar supplemented with tetracycline (50 µg/mL). Clones containing the vector with the insert were added to 5 mL of LB medium supplemented with tetracycline. The culture was incubated at 37 °C for 18 h.

The result was identified by electrophoresis in a 2% agarose gel (Supplementary Figure S2). In addition to antibiotic-resistant screening of the resulting colonies, selective PCR was performed using anti-miR775A primers. The transformed *E. coli HB101* colonies were grown in liquid LB medium to produce biomass and subsequently used to extract the recombinant vector.

### 4.4. Generation of a Transformed A. tumefaciens Line

Plasmid DNA was isolated from *E. coli* bacterial cells using the Cleanup Standard kit (Eurogen, Moscow, Russia) according to the manufacturer’s protocol. The resulting vector containing the target gene was used to transform competent *A. tumefaciens* EHA105 cells by heat shock, following the standard protocol. Transformants were plated on Petri dishes containing YEP agar supplemented with rifampicin (50 µg/mL) and tetracycline (50 µg/mL). Clones containing the vector with the insert were added to 5 mL of liquid YEP medium containing tetracycline (50 µg/mL). The culture was incubated at 28 °C for 72 h.

In addition to antibiotic resistance screening, selective PCR targeting anti-miR775A was performed. PCR electrophoresis was performed on a 2% agarose gel (Supplementary Figure S3).

### 4.5. Generation of a Z. mays Plant Line with miR775A Knockdown

*A. tumefaciens EHA105*, containing the anti-miR775A antisense sequence, was used to generate a *Zea mays* plant line (mir775A) with a knockdown of the corresponding miRNA using the seed-piercing protocol [24]. The plants transformed with the original pBl121 vector without the anti-miR775A construct (WT-Ab) and the WT plants were used as controls. Each test group contained 100 seeds. After transformation, plants were grown hydroponically at 22 °C and a 10 h photoperiod with a light intensity of 90 μmol quanta m^−2^ s^−1^. The T0 generation was used in the experiments.

### 4.6. Creating Hypoxic Conditions

Low oxygen concentrations in the environment were achieved by submerging whole plants in distilled water for 24 h [25,26,27]. The plants not immersed in water were used as the control group. To prevent photosynthetic oxygen production, both plant groups were kept in darkness for 24 h before the experiment, as described earlier [20]. To set up the hypoxia experiment, 50 maize seeds were taken for each plant variant (WT, WT-Ab, and mir775A) to determine enzymatic activity and gene expression.

### 4.7. Isolation of Total mRNA

Guanidine thiocyanate-phenol-chloroform extraction was used to isolate total RNA [28], with LiCl as the precipitant [29]. Qualitative nucleic acid analysis was performed by electrophoresis in a 1% agarose gel and stained with ethidium bromide.

### 4.8. Reverse Transcription

To obtain cDNA, reverse transcription was performed using the MMLV kit (SibEnzyme, Novosibirsk, Russia) with a specific probe developed by us for miR775A and Oligo(dT)_15_ for mRNA. For this purpose, 100 ng of nucleic acids were collected from each fraction. The reverse transcription parameters for mRNA were according to the manufacturer’s recommendations; for miR775A, the following were used: incubation at 16 °C for 30 min, at 42 °C for 30 min, and at 85 °C for 5 min [15].

### 4.9. Real-Time Polymerase Chain Reaction

Real-time polymerase chain reaction with gene-specific primers was performed using the AmpliSence reagent kit (Helicon, Moscow, Russia). To assess transcript-level dynamics, the elongation factor Ef-1α gene served as the reference gene [30,31]. Relative gene transcript levels were determined using the 2^−ΔΔCT^ method [32]. Gene expression levels were calculated relative to the 18S rRNA gene transcript level, which was set to 1. To demonstrate differences between WT and the transgene, the expression of genes encoding mitochondrial and extramitochondrial isoenzymes of aldolase (*Aldo1* and *Aldo* 2), citrate synthase (*Csy1* and *Csy2*) and aconitase (*Aco1* and *Aco2*) was measured.

The nucleotide composition of the primers was for miR775A: forward 5′-CACTGATTCGATGTCTAG-3′; reverse 5′-GTGCAGGGTCCGAGGT-3′. The amplification parameters were as follows: pre-denaturation at 95 °C for 5 min, cycle—95 °C for 30 s, 58 °C for 30 s, 72 °C for 30 s (detection), and final elongation—72 °C for 10 min. For anti-miR775A: forward 5′-TTTAATGGTTCCTGGCACTG-3′; reverse 5′-GTGGAGGAACCTTCGATGTC-3′. The amplification parameters were as follows: pre-denaturation 95 °C for 5 min, cycle—95 °C for 30 s, 55 °C for 30 s, 72 °C for 30 s (detection), final elongation 72 °C for 10 min.

For pri-miR775A: forward 5′-CACTGATTCGATGTCTAG-3′; reverse Oligo-dT_(15)_ [33,34]. For *Aldo1* (LOC100286050): forward—5′-AAGCCCGAAGACACCGATCT-3′; reverse—5′-AAGCAACAGATTTCGCGGTG-3′; for *Aldo2* (LOC100272913): forward—5′-GTGCCAACAACCTCTACGT-3′; reverse—5′-TCTGTTGTGTTGGCACAGG-3′. For M13: forward 5′-GTTGTAAAACGACGGCCAGTG-3′; reverse 5′-AGCGGATAACAATTTCACACAGGA-3′ (Eurogen, Moscow, Russia). Amplification parameters were as follows: pre-denaturation 95 °C for 5 min, cycle—95 °C for 30 s, 58 °C for 30 s, 72 °C for 30 s (detection), final elongation 72 °C for 10 min.

For *Csy1* (LOC100279573): forward 5′-TTTCTTTCGCAGCGGCTCTA-3′, reverse 5′-TGGATCCTTGGGCAAATGCT-3′; for *Csy2* (LOC100275174): forward 5′-AATGGGTTAGCTGGGCCACT-3′, reverse 5′-AGTGGATCCTCGGGCAAATG-3′. Amplification parameters were as follows: pre-denaturation 95 °C, 5 min, cycle—95 °C, 30 s, 58 °C, 30 s, 72 °C, 30 s (detection), final elongation 72 °C, 10 min.

For *Aco1* (LOC100304277): forward 5′-TGGAAGGAGATGCTGTCAGT-3′, reverse 5′-CGTATAGCGCCATCCACATG-3′; for *Aco2* (LOC100281040): forward 5′-CAAGTTCTTCAGCCTTCCGG-3′, reverse 5′-GCAAGGTCTACAACTGCTGG-3′. For *Fum1* (LOC103652742): forward 5′-GATTACTTCGATCATTGAGGT-3′, reverse 5′-ACCAGAACTCGCGGATGTGGC-3′; for *Fum2* (LOC103633973): forward 5′-AGGGCGGTCAGAAGTATGTG-3′, reverse 5′-CAACTTCAAGCTGAATCCTTTCAA-3′. Amplification parameters were as follows: pre-denaturation 95 °C, 5 min, cycle—95 °C, 30 s, 60 °C, 30 s, 72 °C, 30 s (detection), final elongation 72 °C, 10 min.

### 4.10. Measurement of Enzyme Activities and Pyruvate Concentration

The extraction medium for enzymes from maize leaves contained 50 mM Tris-HCl (pH 7.8), 1 mM EDTA, 3 mM MgCl_2_, and 0.3 M sucrose, at a volume of 10 mL per 1 g of plant material. All procedures were carried out at 0–4 °C to prevent enzyme degradation. After sample preparation, the mixture was centrifuged (Eppendorf Centrifuge 5805R, Sigma-Aldrich, St. Louis, MO, USA) for 5 min at 3000× *g* at 4 °C, and the supernatant was used for further studies. To isolate the cytosolic and mitochondrial fractions, differential centrifugation was performed at 14,000× *g* for 20 min. The supernatant was used to determine the activity of cytoplasmic enzymes. The pellet was dissolved in 1 mL of sucrose-free isolation medium and used to measure PDH activity. The activities of studied enzymes were measured using an SF-2000 spectrophotometer (OKB Spektr, St. Petersburg, Russia). The amount of enzyme that formed 1 μmol of the corresponding product in 1 min at 25 °C was taken as a unit of enzymatic activity. All chemicals were from Sigma-Aldrich (St. Louis, MO, USA).

Fructose-1,6-bisphosphate aldolase (EC 4.1.2.13) activity was measured at 240 nm by the formation of aldehyde in 50 mM Tris-HCl, pH 7.5, 1 mM EDTA, 3.5 mM hydrazine sulfate, 1.2 mM fructose-1,6-bisphosphate [35].

Lactate dehydrogenase (EC 1.1.1.27) activity was determined as the rate of NAD^+^ reduction at 340 nm. The reaction medium contained 50 mM Tris-HCl buffer, pH 7.4, 0.5 mM NAD^+^, and 2.5 mM sodium lactate [36].

Pyruvate dehydrogenase (EC 1.2.4.1) activity was determined as the rate of NADH oxidation at 340 nm. The reaction medium contained 50 mM Tris-HCl buffer, pH 8.0, 2.5 mM NAD^+^, 0.2 mM thiamine chloride, 0.13 mM coenzyme A, 2.5 mM cysteine, and 2 mM sodium pyruvate [37].

Pyruvate kinase (EC 2.7.1.40) activity was measured using an auxiliary reaction in the presence of one enzyme unit of PDH. The reaction medium contained 50 mM Tris-HCl buffer, pH 7.7, 2.5 mM NAD^+^, 0.2 mM thiamine chloride, 0.13 mM coenzyme A, 2.5 mM cysteine, 3 mM phosphoenolpyruvate, 1 mM MgCl_2_, and 1 unit of PDH (Sigma Aldrich, St. Louis, MO, USA).

The concentration of pyruvate was determined by an enzymatic method with LDH at 340 nm using the commercial kit PYRUVATE UV-ABRIS+ (NPF ABRIS+, St. Petersburg, Russia).

### 4.11. Statistical Data Processing

A total of three plant transformations were conducted. Each transformed plant variant was initially analyzed for morphological parameters and transformation control, followed by a study of enzyme activity, gene expression, and pyruvate content. The data were subjected to two-way analysis of variance (ANOVA) using STATISTICA data analysis software version 9.0 (Statsoft Wipro, East Brunswick, NJ, USA). The results are presented as mean values and standard deviations (SD). Statistically significant differences are discussed at *p* < 0.05 [38]. Asterisks represent significant differences according to one-way ANOVA analysis at *p* < 0.05 (Tukey’s multi-comparison). Electrophoregrams and the images of plants represent the data from typical experiments repeated three to four times.

## 5. Conclusions

Using an anti-miR775A construct, we generated a knockdown line of *Z. mays* plants with reduced levels of the hypoxia-dependent miR775A. Morphological changes in the modified plants and quantification of mature miR775A demonstrated the effectiveness of the method of knockdown plant generation. Reduced levels of mature miR775A in the leaves of 14-day-old knockdown maize plants delayed seedling development, leaf blade formation, and leaf unfolding. Biochemical changes include a suppression of the glycolytic pathway, leading to a decrease in pyruvic acid levels in leaf cells. The lack of hypoxic increase in LDH in mir775A knockdown maize plants may explain the slow seedling development, as the limitation of NADH regeneration in this reaction suppresses glycolysis, the primary energy pathway under oxygen deficiency. An analysis of several key enzymes involved in cellular energy and biosynthetic metabolism clearly indicates the role of miRNA775A in the reconfiguration of metabolic flows, which can be further studied using metabolic flux analysis.

## Figures and Tables

**Table 1:** Length of maize seedlings of control and experimental samples.

Day	Length of Seedlings, cm		
	WT	WT-Ab	*mir775A*
2	0.59 ± 0.06	0.51 ± 0.04	0.56 ± 0.07
5	4.71 ± 0.25	4.83 ± 0.30	***3.20 ± 0.14***
10	9.68 ± 0.31	9.34 ± 0.41	***5.31 ± 0.25***

Abbreviations: WT, wild-type maize plants; WT-Ab, maize plants modified with the original *A. tumefaciens EHA105* line; mir775A, knockdown maize plants. Bold and italic fonts indicate the statistical significance of differences in seedling length.

## Data Availability

The original contributions presented in this study are included in the article/Supplementary Materials. Further inquiries can be directed to the corresponding author.

## Supplementary Materials

The following supporting information can be downloaded at: https://www.mdpi.com/article/10.3390/ijms27072943/s1.

## Abbreviations

miRNA	microRNA
mir775A	maize line with the knockdown of microRNA775A
pri-miR	the initially transcribed long primary transcripts from the precursor gene of the corresponding microRNA
LDH	lactate dehydrogenase
PDH	pyruvate dehydrogenase
WT	wild type
WT-Ab	the plants transformed with the original pBl121 vector without the anti-miR775A construct
