# Supplementary material for: Generation and Characterization of a Genetically Modified Zea mays Line with a Knockdown of Hypoxia-Dependent microRNA775A
Source: Int J Mol Sci. 2026 Mar 24;27(7):2943. doi: 10.3390/ijms27072943 (PMC13073709; doi:10.3390/ijms27072943)
Supplement: Supplementary file 1 [file ijms-27-02943-s001.zip › ijms-4157780-supplementary.pdf]

Supplementary materials to the paper "Generation and characterization of a genetically modified *Zea mays* line with a knockdown of hypoxia-dependent microRNA775A" by Dmitry N. Fedorin, Anna E. Khomutova, Alexander T. Eprintsev and Abir U. Igamberdiev

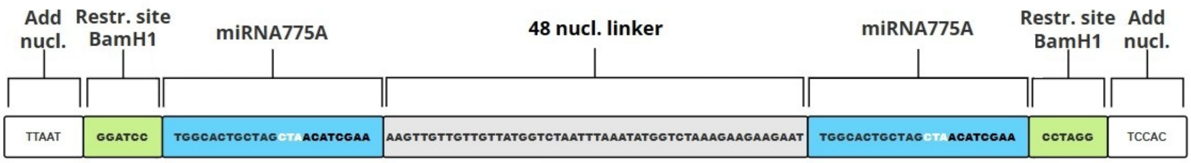

Supplementary Figure S1. Schematic diagram of the anti-miR construct for microRNA775A. Abbreviations: Add. nucl., additional 5 nucleotides at the 5' and 3'-ends; Restr. site BamH1, BamH1 endonuclease restriction site; CTA, hairpin region for degradation between the 10th and 11th nucleotides of microRNA775A.

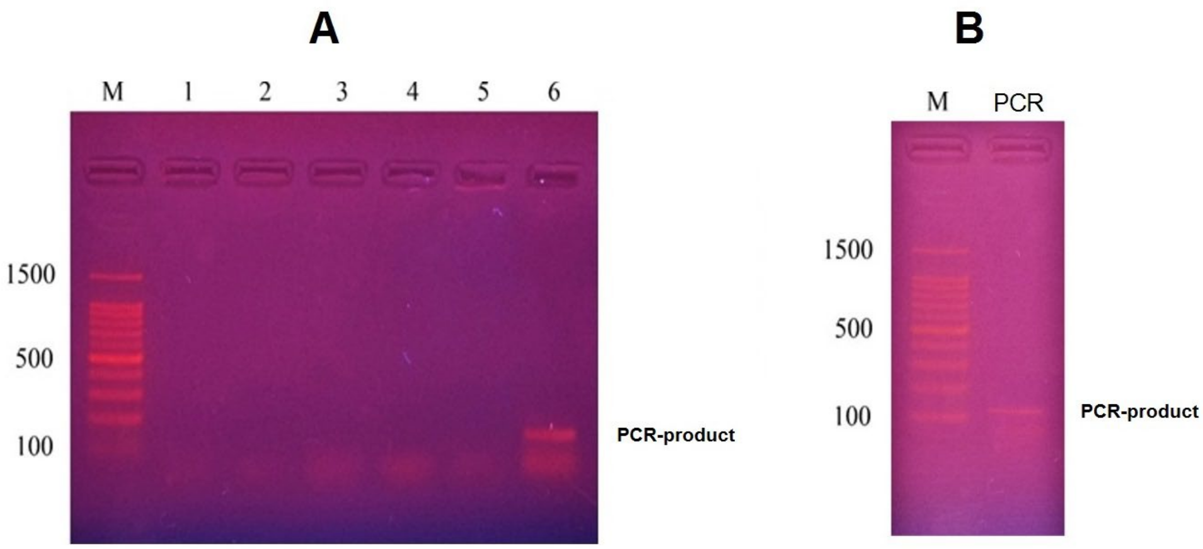

Supplementary Figure S2. Transformation of *E. coli HB101* and *A. tumefaciens EHA105*. A – Screening of the resulting *E. coli* colonies by selective-specific PCR. B – Screening of the resulting colonies of modified *A. tumefaciens EHA105* by selective-specific PCR.

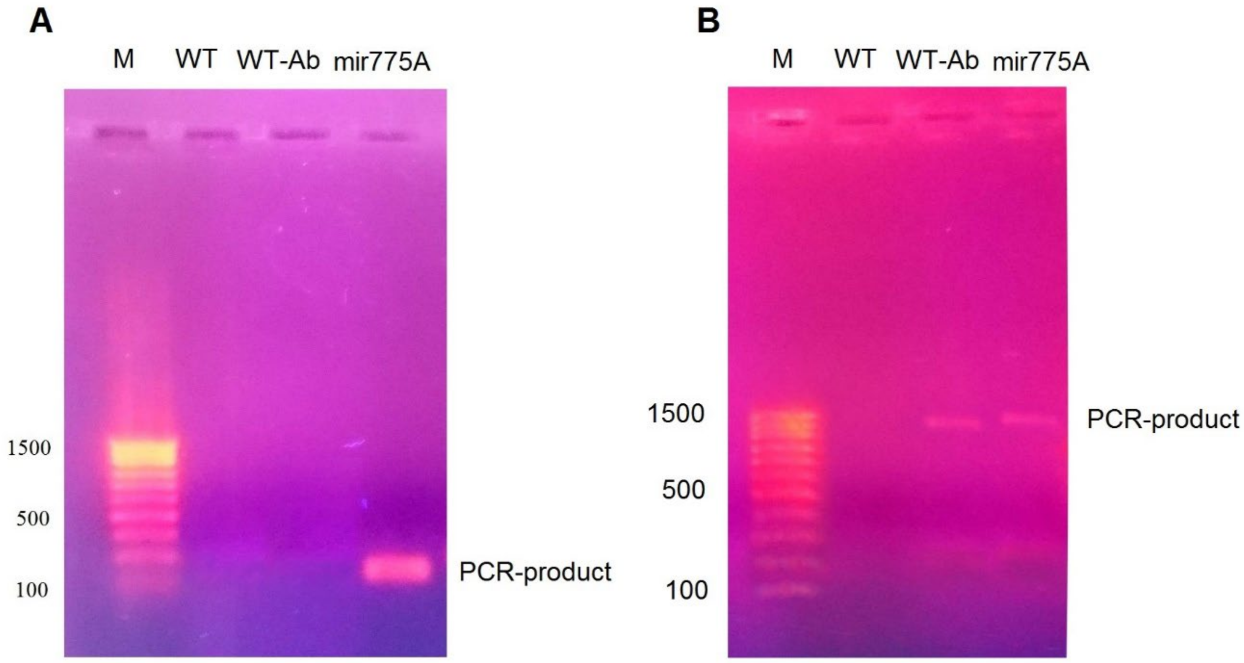

Supplementary Figure S3. DNA amplification results. A – DNA amplification product of wild-type (WT) maize, modified by the original *A. tumefaciens EHA105* line (WT-Ab), and knockdown plants for microRNA775A (mir775A) with M13 primers. B – DNA amplification product of wild-type (WT) maize, modified with the original *A. tumefaciens EHA105* line (WT-Ab), and knockdown plants for microRNA775A (mir775A) with primers to the anti-miR775A construct. M – DNA length markers.
